# Case Report: Novel mutations in two patients with *MED13L*-related intellectual disability highlighting the importance of genetic counseling

**DOI:** 10.3389/fgene.2025.1669849

**Published:** 2025-12-01

**Authors:** Hanyu Cao, Tiantian He, Jing Wang, Cong Zhou, Xing Wei, Xuemei Zhang

**Affiliations:** 1 Department of Medical Genetics and Prenatal Diagnosis Center, West China Second University Hospital, Sichuan University, Chengdu, China; 2 Key Laboratory of Birth Defects and Related Diseases of Women and Children (Sichuan University), Ministry of Education, Chengdu, China

**Keywords:** *MED13L*, genetic counseling, recurrence risk, intellectual disability, novel mutation, unreported symptoms

## Abstract

*MED13L*-related intellectual disability (ID) (impaired intellectual development and distinctive facial features with or without cardiac defects, OMIM: 616789) is a neurodevelopmental condition characterized by intellectual disability, hypotonia, motor delay, and remarkable speech delay. We report two novel cases, each harboring a novel pathogenic *MED13L* variant, who presented with additional, previously unreported features: oligospermia in a 32-year-old male proband and oligohydramnios and hematuria in a 6-year-old female proband, thereby expanding the known phenotypic spectrum. The study underscores the value of genetic testing and counseling, exemplified by the successful prenatal diagnosis and birth of an unaffected child in the second family. In addition, we reviewed previous literature with respect to phenotypic and genetic information. The literature reviewed here may potentially provide information for assessing clinical symptoms and genetic counseling. This study also highlights the importance of preconception genetic counseling for couples with suspected genetic disease.

## 1 Introduction

The *MED13L* gene maps on chromosome 12q24.21 and consists of 31 exons and, as its name suggests, encodes a subunit protein of the large mediator complex, which is involved in gene transcription mediated by transcription factors and RNA polymerase II (Lewis and Reinberg, 2003). Its correlation with intellectual disability (ID) and congenital heart defects was first reported in a girl with a *de novo MED13L*-disrupting translocation two decades ago by Muncke et al. (2003)). Although the role of *MED13L* in congenital heart disease has been reported by several research studies, increasing clinical and experimental evidence has successively demonstrated the vital role of *MED13L* in the regulation of the neural crest cells and neurogenesis, along with its morphological and functional impacts on cortical neurons, which may exert great influence on intellectual development (Muncke et al., 2003; Angus and Nevins, 2012; Asadollahi et al., 2017; Hamada et al., 2023). Rather, the initially described complex disorder has been recently redefined as a syndromic ID that encompasses a distinctive set of features, including dysmorphic facial features, ID, and speech impairment (impaired intellectual development and distinctive facial features with or without cardiac defects; OMIM: 616789), with variable penetrance for congenital heart defects, inherited in an autosomal dominant manner (Adegbola et al., 2015).

To date, only approximately 100 instances with definite or suspected *MED13L*-related intellectual disability have been documented in published literature. Previously reported cases with missense variants appear to display a more severe clinical phenotype than protein-truncating variants, while copy number gain of the gene results in a milder phenotype with learning disability and moderate facial dysmorphism (Asadollahi et al., 2014; Asadollahi et al., 2013).

In this study, we report two probands, each with a *de novo MED13L* mutation detected by exome sequencing, which had not been previously reported in the literature. The two cases we presented also underscore the importance of preconception genetic counseling, especially for families with suspected genetic disease. In addition, we further review the literature for previously reported patients and expand the phenotypic and genetic spectrum of *MED13L*-related intellectual disability.

## 2 Case presentation

### 2.1 Proband 1

#### 2.1.1 Case presentation

The proband was a 32-year-old man referred to our clinic due to a two-year history of primary infertility with his wife who was diagnosed with dwarfism at 12 years old and later was found to harbor a novel variant (c.1133T>C (p.F378S) (NM_004560.4)) in ROR2 gene presenting at homozygous state with her distinctive features (hypertelorism, prominent eyes, wide palpebral fissures, broad and depressed nasal bridge, short upturned nose, anteverted nares, tented upper lip, broad and triangular mouth, brachydactyly, hypoplastic nails, and fifth finger clinodactyly) consistent with Robinow syndrome. Family-based segregation analysis showed that both his wife’s siblings and her parents were ROR2 c.1133T>C heterozygous carriers. Since his wife desired pregnancy and came to our hospital, we recommended that her husband (the proband) undergo a comprehensive examination in our hospital.

**FIGURE 1:**
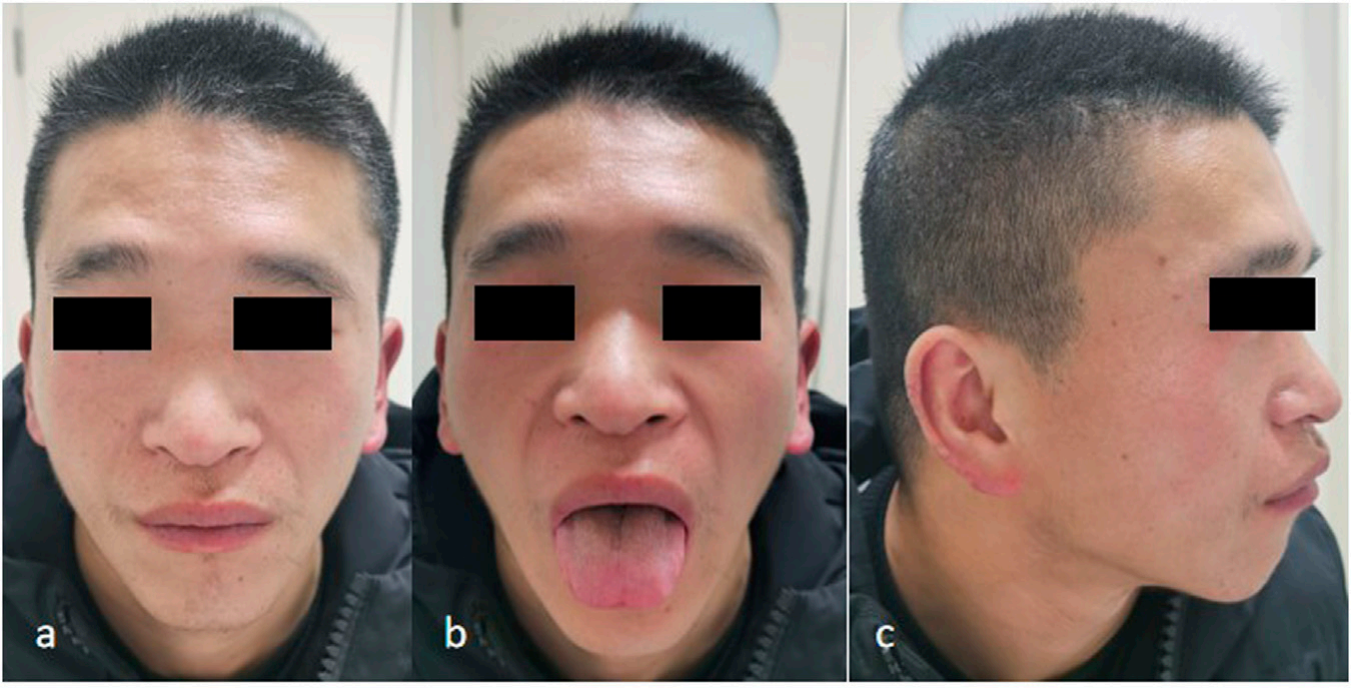
Clinical features in proband 1. **(a)** Triangular face, bulbous nasal tip, **(b)** macroglossia, **(c)** low nasal root, and relatively low-set ears, which are characteristic facial features associated with *MED13L*-related syndrome.

The prenatal and perinatal history could not be accurately obtained. Occipitofrontal circumference at birth was not measured. The details regarding his growth and development during childhood are not available. From an early age, the patient had a global developmental delay; he attended kindergarten for 2 years and did not receive special or formal education. Recent intelligence testing indicated significant cognitive impairment at the level of moderate intellectual disability. Upon inspection, it was observed that the patient exhibited characteristic facial features with a triangular face, hypertelorism, low nasal root, bulbous nasal tip, macroglossia, and poor speech, along with severe communication impairment (Figure 1). According to the parents, there was no known history of cardiac disease in the patient, though they were unable to provide any prior medical records or reports of cardiac investigations. Previous medical examination reports, from an andrological outpatient clinic, showed normal external genitalia, and a semen examination, performed at another hospital, demonstrated oligospermia.

#### 2.1.2 Investigations

**FIGURE 2:**
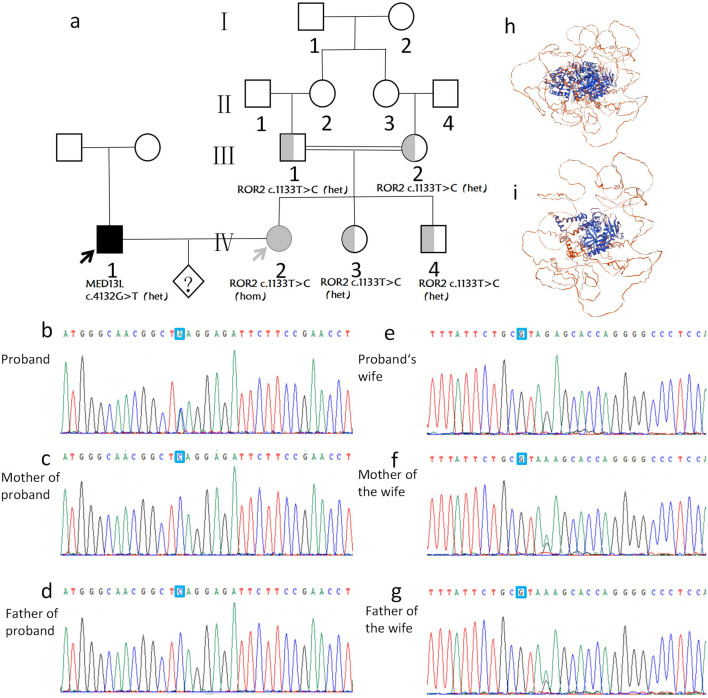
The family pedigree **(a)** of proband 1 and Sanger sequencing results **(b–g)**. A novel *de novo* c.4132G>T (p.E1378*) (NM_015335.5; ClinVar: VCV002497747.1) variant of the MED13L gene (OMIM: 608771) was found in the proband 1 **(b)** but not in the proband 1’s parents **(c,d)**. A ROR2 c.1133T>C (p.F378S) (NM_004560.4; ClinVar: VCV002446722.1) was present at homozygous state in the proband’s wife **(e)** and heterozygous state in the individual’s parents **(f,g)**. Modeled 3D structure demonstrating the pathogenic mechanism of novel MED13L mutations. Wild-type MED13L protein structure **(h)** is shown for comparison. The mutant structure **(i)** corresponding to the c.4132G>T (p.E1378*) variant reveals a premature truncation, leading to the loss of critical functional domains.

Exome sequencing was conducted, and a novel *de novo* c.4132G>T (p.E1378*) (NM_015335.5) variant of the *MED13L* gene (OMIM: 608771) was found in the proband. According to the ACMG 2015 guidelines, the c.4132G>T mutation was classified as pathogenic (PVS1, PS2, and PM2) (Richards et al., 2015). Figure 2 illustrates this family’s pedigree (a) and the results of Sanger sequencing (b–g). This mutation results in an amino acid substitution of glutamic acid for a stop codon at position 1,378 of the protein (E1378X), leading to the loss of the MedPIWI domain and the highly conserved C-terminal domain, which potentially affects the 3D structure of the MED13 protein (h–i).

#### 2.1.3 Genetic counseling and outcome

Upon receiving the genetic diagnosis, the parents and wife of proband 1 expressed significant anxiety and concerns regarding their future reproductive options. A comprehensive genetic counseling spanning multiple sessions to address their ongoing questions was performed to provide guidance on fertility and evaluate the recurrence risk. Given the heterozygous genotype of the proband (IV1 subject) and the autosomal dominant inheritance model of *MED13L*, each offspring had a 50% chance of being affected. With regard to the wife (IV2 subject) with homozygous *ROR2* alleles and her husband, who did not carry any *ROR2* variants, all offspring will be carriers, but none will be affected. Due to their 2-year history of infertility, we suggested that they undergo infertility evaluation in our reproductive department, and prenatal diagnosis was recommended for this couple. The parents and his wife were fully informed and reported a better understanding of the condition, stating that their reproductive decision-making will involve discussions with their families. Despite our efforts for follow-up, they were subsequently lost to follow-up, and the outcome of their reproductive decision-making is unknown.

### 2.2 Proband 2

#### 2.2.1 Case presentation

The patient was a girl aged 6 years 11 months who was born at full-term by normal delivery, and the pregnancy was complicated by late oligohydramnios, without amniocentesis for prenatal diagnostic testing. The neonatal screening test was normal. She could lift her head unstably at 6 months, required assistance to sit at 10 months, and was unable to sit alone until she was one year old. Karyotyping revealed a normal female karyotype of 46,XX, and her electroencephalography (EEG) result was negative at 10 months old. Motor development was evaluated using the Peabody Developmental Motor Scales-2 (PDMS-2) at 14 months old with a GMQ quotient of 83, an FMQ quotient of 73, and a TMQ quotient of 77, indicating significant motor delay, with fine motor skills being an area of particular weakness. Her expressive language development was also delayed, and she presented global developmental delay at the age of 1 year and 3 months. Her developmental scores at 2 years old, assessed by the Griffiths Development Scales-Chinese (GDS-C), were equivalent to those of 12-to-14-month-old skills. This significant delay is consistent with the profound global developmental delay that is a hallmark of *MED13L*-related disorder. She underwent inpatient evaluation at 3 years old due to recurrent hematuria persisting over 1 month, with provisional diagnoses of hereditary nephropathy or IgA nephropathy. The echocardiography demonstrated an atrial septal defect, mild tricuspid regurgitation, and a persistent Eustachian valve when she was 5 years old. An MRI of the brain revealed abnormal patchy signal intensity in the periventricular white matter and centrum semiovale, multiple patchy and strip-shaped shadows in the posterior horn of the left lateral ventricle, and dilatation of both lateral ventricles.

#### 2.2.2 Investigations

**FIGURE 3:**
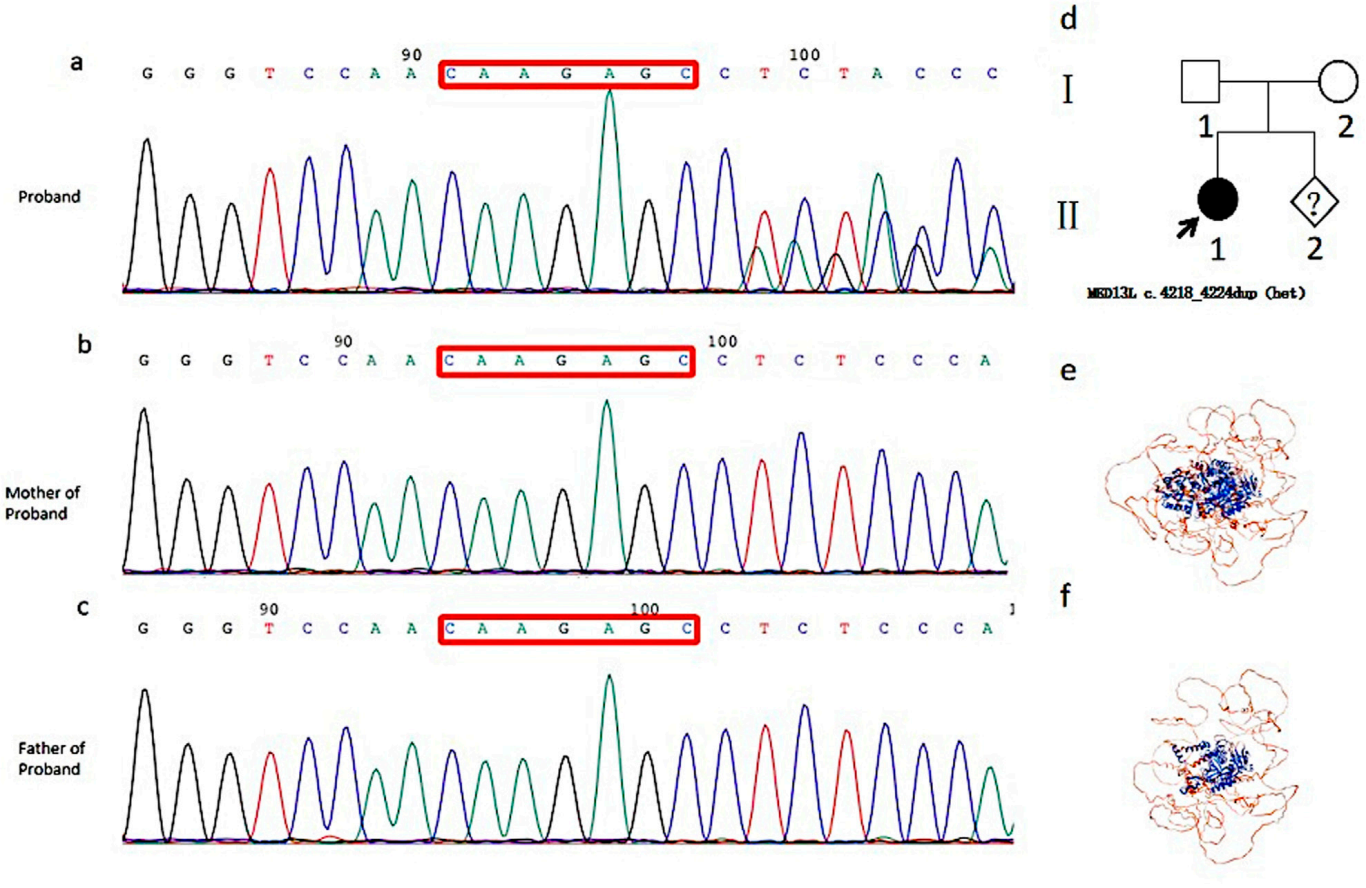
Sanger sequencing results **(a–c)** of proband 2 and the family pedigree **(d)**. A novel *de novo* c.4218_4224dup (p.L1409fs) (NM_015335.4; ClinVar: VCV004526649.1) variant of the MED13L gene (OMIM: 608771) was found in proband 2 **(a)** but not in the proband 2’s parents **(b, c)**. The modeled 3D structure of the wild-type **(e)** and mutant **(f)** MED13L protein illustrates that the p.L1409fs variant introduces a premature termination codon, predicting a truncated protein and loss of function.

After the analysis of the exome sequencing data, a novel *de novo* c.4218_4224dup (p.L1409fs) variant in exon 19 of the *MED13L* gene (NM_015335.4) co-segregating with the phenotype was identified, which was confirmed using Sanger sequencing. The variant was not reported in gnomAD, ExAC, ClinVar, or other databases. Therefore, c.4218_4224dup in this patient was classified as pathogenic according to the American College of Medical Genetics and Genomics guidelines (ACMG) (Richards et al., 2015) (PVS1, PS2, and PM2). Figure 3 shows the results of Sanger sequencing (a–c) and this family’s pedigree (d). It is located near the MID domain of the MedPIWI module, which is the core globular domain of the MED13 protein, ultimately introducing a premature stop codon and truncating the MED13L protein at amino acid 1,409 (e–f).

#### 2.2.3 Genetic counseling and outcome

Initially, the parents exhibited considerable distress and uncertainty upon receiving the diagnosis. Sequential comprehensive genetic counseling was conducted with the aim of explaining the results and was focused on two key aspects: 1) the management plan for the affected child, including anticipated developmental delays, the need for early intervention services, and recommendations for regular cardiac surveillance, and 2) providing fertility guidance since her mother was pregnant again. After learning that prenatal diagnosis was available for the present pregnancy based on the identified genetic variant, they felt more equipped and empowered. Given the *de novo* nature of the mutation and the autosomal dominant inheritance model of *MED13L*, we suggested that her mother receive a molecular prenatal diagnosis by amniocentesis to rule out gonadal mosaicism and prevent the recurrence of another affected child, despite the low risk of recurrence. Amniocentesis was conducted at 18 weeks of gestation, and a molecular prenatal diagnosis of the genomic DNA extracted from the amniotic fluid was performed for the frameshift mutation, for which the fetus was of the wild-type, leading to the decision to continue the pregnancy. A third-trimester fetal brain MRI revealed unremarkable intracranial findings. The subsequent successful delivery of a healthy, full-term infant brought the parents great relief and joy, significantly alleviating their initial anxiety.

## 3 Discussion

The *MED13L* gene comprises 31 exons and encodes a subunit of the mediator complex, which is an essential transcriptional coactivator implicated in RNA polymerase II-directed transcription. Previous studies reported that *MED13L*-missense variants cluster in exons 15–17 and 25–31, while truncating variants were clustered in exons 10 and 29 (Smol et al., 2018). The *MED13L* gene was classified as haploinsufficient in the ClinGen database, which means the mechanism of disease is haploinsufficiency, although previous studies suggested that patients harboring missense variants appear to display a more severe phenotype (Bessenyei et al., 2022). Previous reported cases with missense variants appear to display a more severe clinical phenotype than protein-truncating variants, while copy number gain of the gene results in a milder phenotype with learning disability and moderate facial dysmorphism. In this study, we presented two new patients, each with a *de novo* truncating mutation (c.4132G>T, p.E1378*, and c.4218_4224dup, p.L1409fs) variant. Both variants were in exon 19 of the *MED13L* gene and were predicted to likely trigger nonsense-mediated decay or post-translational degradation. Although *MED13L* appears to be one of the most frequently mutated syndromic ID gene, available data are scarce on the correlation between genotypes and phenotypes in this condition, and the gene–disease validity of *MED13L* with congenital heart disease has been classified as limited in the ClinGen database.

*MED13L*-related intellectual disability is a syndromic intellectual disability caused by *MED13L* gene mutations inherited in an autosomal-dominant pattern (Adegbola et al., 2015; Bessenyei et al., 2022). Patients with *MED13L*-related intellectual disability have a broad range of clinical manifestations. We reviewed the published literature on the topic and obtained all the available information on reported patients, while nine were excluded due to a lack of description of clinical features (Muncke et al., 2003; Asadollahi et al., 2017; Hamada et al., 2023; Adegbola et al., 2015; Asadollahi et al., 2013; Smol et al., 2018; Bessenyei et al., 2022; Aoi et al., 2019; Cafiero et al., 2015; Caro-Llopis et al., 2016; Carvalho et al., 2021; Chang et al., 2022; Codina-S et al., 2015; Dawidziuk et al., 2021; Gilissen et al., 2014; Gordon et al., 2018; Iglesias et al., 2014; Iossifov et al., 2012; Ji et al., 2020; Jimenez-Romero et al., 2018; Mainali et al., 2023; Martinez et al., 2017; Mullegama et al., 2017; Musante et al., 2004; Najmabadi et al., 2011; Park et al., 2022; Sabo et al., 2020; Shahid et al., 2024; Siavriene et al., 2023; Tian et al., 2021; Torring et al., 2019; Utami et al., 2014; van Haelst et al., 2015; Yamamoto et al., 2017; Yi et al., 2020). Among them, there were 21 patients harboring likely truncating/LOF variants and 70 harboring missense variants of *MED13L*. Their clinical features are listed in Table 1 and detailed in Supplementary Table S1. All patients with available information had ID (91/91, 100%) and speech delay (86/86, 100%), while 83 patients had motor delay (83/84, 99%). Fifty-eight patients had hypotonia (58/81, 72%). Anomalies of hands and feet (38/80, 48%) and cerebral MR abnormalities (30/64, 47%) were observed in slightly less than half of the patients. Ophthalmological abnormalities were described in 31 of 80 (39%) patients, autistic features in 22 of 78 (28%) patients, and initially described complex congenital heart defects in only 20 of 80 (25%) patients. With respect to facial dysmorphisms, bulbous nasal tip (56/74, 76%), depressed/broad nasal bridge (23/42, 55%), open mouth appearance (44/72, 61%), and low-set ears (21/39, 54%) were observed in more than half of the patients. Less common features include upslanting palpebral fissures (29/74, 39%), horizontal eyebrows (8/38, 21%), macrostomia (16/37, 43%), macroglossia, bitemporal narrowing, and large ears, which were present in 19%–43% of the patients. Our review of the available literature suggests that hypotonia, ophthalmological abnormalities, motor delay, abnormalities in cerebral MR, and autistic features may be more common in patients carrying missense variants than in patients with truncating variants; however, this observation requires confirmation by future systematic reviews with larger sample sizes. Nevertheless, all dysmorphic facial features except low-set ears were more frequently observed in patients with likely truncating variants.

**TABLE 1:** Clinical features of 91 previously reported patients with mutations in *MED13L*.

		Missense, No of patients (%)	Truncating, No of patients (%)	Total No of patients (%)
Clinical features	ID	21/21 (100%)	70/70 (100%)	91/91 (100%)
	Speech delay	18/18 (100%)	68/68 (100%)	86/86 (100%)
	Motor delay	18/18 (100%)	65/66 (98%)	83/84 (99%)
	Anomalies hand/feet	5/16 (31%)	33/64 (52%)	38/80 (48%)
	Hypotonia	14/17 (82%)	44/64 (69%)	58/81 (72%)
	Congenital heart defects	4/17 (24%)	16/63 (25%)	20/80 (25%)
	Ophthalmological	7/16 (44%)	24/64 (38%)	31/80 (39%)
	Anomalies cerebral MR	8/15 (53%)	22/49 (45%)	30/64 (47%)
	Autistic features	9/16 (56%)	13/62 (21%)	22/78 (28%)
Dysmorphic features	Bulbous nasal tip	9/17 (53%)	47/58 (81%)	56/74 (76%)
	Depressed/broad nasal bridge	2/7 (29%)	21/35 (60%)	23/42 (55%)
	Upslanting palpebral fissures	3/16 (19%)	26/58 (45%)	29/74 (39%)
	Horizontal eyebrows	1/6 (17%)	7/32 (22%)	8/38 (21%)
	Macrostomia	1/6 (17%)	15/31 (48%)	16/37 (43%)
	Macroglossia	1/6 (17%)	11/34 (32%)	12/40 (30%)
	Open mouth appearance	9/15 (60%)	35/57 (61%)	44/72 (61%)
	Low set ears	6/7 (86%)	15/32 (47%)	21/39 (54%)
	Brachycephaly	0/5 (0%)	7/31 (23%)	7/36 (19%)
	Bitemporal narrowing	1/7 (14%)	10/35 (29%)	11/42 (26%)
	Large ears	1/6 (17%)	6/30 (20%)	7/36 (19%)

Patient 1 exhibits moderate ID, severe language impairment, and distinctive facial features and is able to engage in routine manual labor. The prominent symptoms of the second proband are delayed psychomotor development, poor speech, and cardiac anomalies, which are in accordance with previously reported clinical manifestations. However, the details regarding the first proband’s prenatal/perinatal history and childhood development are a notable limitation. The first proband had no reported history of cardiac issues based on parental recall, while the second proband suffered from complex cardiac malformations. This phenotypic variability highlights the variable penetrance of cardiac manifestations associated with *MED13L* mutations (van Haelst et al., 2015). Additionally, both the symptoms of oligospermia in the first proband and oligohydramnios and hematuria in the second proband have not been previously described in *MED13L*-mutation patients despite rare reports of other genitourinary anomalies such as cryptorchidism, micropenis, and renal agenesis (Adegbola et al., 2015; Caro-Llopis et al., 2016). These novel findings align with the gene’s emerging role in multi-system development, but they remain speculative without functional validation and highlight a clear direction for future research, including larger patient cohorts to establish the true prevalence of these features and functional investigations to examine the role of *MED13L* in the developing kidneys and reproductive tract. Furthermore, oligohydramnios may represent a prenatal indicator of potential renal impairment in fetuses with *MED13L* mutations, serving as a clinical clue for early detection. However, confirming this association requires further case analysis across diverse ethnic populations (Yi et al., 2020). The MRI examination of case 2 showed anomalies that resembled inherited metabolic disease; however, most of the reported MRI abnormalities were nonspecific (Yi et al., 2020).

Moreover, comprehensive preconception counseling has critical reproductive implications for both families, which allows for appropriate medical management. For the first family, preconception genetic counseling could be difficult due to unintended pregnancy, an incomplete family history of genetic diseases, egg or sperm donations, financial barriers, and patients’ distrust, which may lead to crucial short- and long-term consequences. Other rare conditions, such as distinct genetic diseases in a couple, may also pose a challenge for preconception genetic counseling because a non-carrier partner of a genetic disease may be a patient of another genetic disease. This is also the first case presenting experiences of a couple who attend preconception genetic counseling, each with a genetic disease caused by novel mutations. Although comprehensive follow-up data were unavailable in this case, the challenges in maintaining long-term engagement with families after genetic counseling highlight the need for integrated care models and digital health tools to support continuity of care in rare disease populations. For the second family, despite the *de novo* variant, a prenatal diagnosis is still needed to rule out the possibility of gonadal mosaicism and prevent another affected child since parental mosaicism increases the recurrence risk. Though the risk could not be accurately calculated, a recurrence risk of <1% is usually used in genetic counseling regarding families of children with *de novo* disease-causing variants. Comprehensive genetic counseling is a critical component of individualized management and fertility planning for families such as our case 2.

The novel mutations reported here will enrich the *MED13L* mutation databases and significantly reinforce the need for delineation of MRFACD clinical phenotypes. Moreover, this paper highlighted the importance of preconception genetic counseling for couples when either or both partners are suspected of having a genetic disease and for families of children with *de novo* genetic variants. In addition, we reviewed previous literature with respect to phenotypic and genetic information of patients with *MED13L*-related intellectual disability. To date, the broad spectrum of *MED13L* variants and phenotypes remains elusive. Further case series, cohort studies, and functional investigations are required to clarify the genotype–phenotype correlation for specific *MED13L* features.

## Data Availability

The datasets presented in this study are publicly available in the online repository ClinVar. The accession numbers are VCV002497747.1, VCV002446722.1, and VCV004526649.1.
