# Correction: Case Report: Novel mutations in two patients with *MED13L*-related intellectual disability highlighting the importance of genetic counseling

**DOI:** 10.3389/fgene.2026.1839890

**Published:** 2026-04-29

**Authors:** Hanyu Cao, Tiantian He, Jing Wang, Cong Zhou, Xing Wei, Xuemei Zhang

**Affiliations:** 1 Department of Medical Genetics and Prenatal Diagnosis Center, West China Second University Hospital, Sichuan University, Chengdu, China; 2 Key Laboratory of Birth Defects and Related Diseases of Women and Children (Sichuan University), Ministry of Education, Chengdu, China

**Keywords:** MED13L, genetic counseling, recurrence risk, intellectual disability, novel mutation, unreported symptoms

There was a mistake in Figure 2 as published. In the pedigree diagram, the symbol representing a heterozygous individual (IV4) was mistakenly shaded as fully filled (indicating homozygosity), instead of half-filled. The corrected Figure 2 appears below.

There was a mistake in the caption of Figure 2 as published. The gene accession number was erroneously written as “NM_4560.4” and clinvar IDs have not been added. The corrected caption of Figure 2 appears below.

**FIGURE 2:**
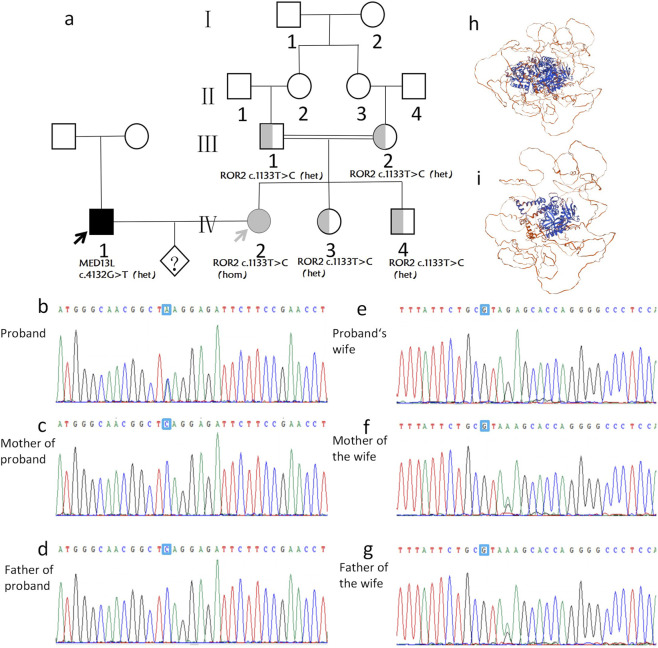
The family pedigree **(a)** of proband 1 and Sanger sequencing results **(b–g)**. A novel *de novo* c.4132G>T (p.E1378*) (NM_015335.5; ClinVar: VCV002497747.1) variant of the MED13L gene (OMIM: 608771) was found in the proband 1 **(b)** but not in the proband 1’s parents **(c,d)**. A ROR2 c.1133T>C (p.F378S) (NM_004560.4; ClinVar: VCV002446722.1) was present at homozygous state in the proband’s wife **(e)** and heterozygous state in the individual’s parents **(f,g)**. Modeled 3D structure demonstrating the pathogenic mechanism of novel MED13L mutations. Wild-type MED13L protein structure **(h)** is shown for comparison. The mutant structure **(i)** corresponding to the c.4132G>T (p.E1378*) variant reveals a premature truncation, leading to the loss of critical functional domains.

There was a mistake in Table 1 as published. The terms “Bulboussal tip”, “Depressed/broad sal bridge”, and “Upslanting palp fissures” should be corrected to “Bulbous nasal tip”, “Depressed/broad nasal bridge”, and “Upslanting palpebral fissures”, respectively. The corrected Table 1 appears below.

**TABLE 1:** Clinical features of 91 previously reported patients with mutations in MED13L.

​	​	Missense,No of patients (%)	Truncating,No of patients (%)	Total No of patients (%)
Clinical features	ID	21/21 (100%)	70/70 (100%)	91/91 (100%)
	Speech delay	18/18 (100%)	68/68 (100%)	86/86 (100%)
	Motor delay	18/18 (100%)	65/66 (98%)	83/84 (99%)
	Anomalies hand/feet	5/16 (31%)	33/64 (52%)	38/80 (48%)
	Hypotonia	14/17 (82%)	44/64 (69%)	58/81 (72%)
	Congenital heart defects	4/17 (24%)	16/63 (25%)	20/80 (25%)
	Ophthalmological	7/16 (44%)	24/64 (38%)	31/80 (39%)
	Anomalies cerebral MR	8/15 (53%)	22/49 (45%)	30/64 (47%)
	Autistic features	9/16 (56%)	13/62 (21%)	22/78 (28%)
Dysmorphic features	Bulbous nasal tip	9/17 (53%)	47/58 (81%)	56/74 (76%)
	Depressed/broad nasal bridge	2/7 (29%)	21/35 (60%)	23/42 (55%)
	Upslanting palpebral fissures	3/16 (19%)	26/58 (45%)	29/74 (39%)
	Horizontal eyebrows	1/6 (17%)	7/32 (22%)	8/38 (21%)
	Macrostomia	1/6 (17%)	15/31 (48%)	16/37 (43%)
	Macroglossia	1/6 (17%)	11/34 (32%)	12/40 (30%)
	Open mouth appearance	9/15 (60%)	35/57 (61%)	44/72 (61%)
	Low set ears	6/7 (86%)	15/32 (47%)	21/39 (54%)
	Brachycephaly	0/5 (0%)	7/31 (23%)	7/36 (19%)
	Bitemporal narrowing	1/7 (14%)	10/35 (29%)	11/42 (26%)
	Large ears	1/6 (17%)	6/30 (20%)	7/36 (19%)

There was a mistake in the caption of Figure 3 as published. The clinvar ID have not been added. The corrected caption of Figure 3 appears below.

“Sanger sequencing results (a–c) of proband 2 and the family pedigree (d). A novel *de novo* c.4218_4224dup (p.L1409fs) (NM_015335.4; ClinVar: VCV004526649.1) variant of the MED13L gene (OMIM: 608771) was found in proband 2 (a) but not in the proband 2’s parents (b, c). The modeled 3D structure of the wild-type (e) and mutant (f) MED13L protein illustrates that the p.L1409fs variant introduces a premature termination codon, predicting a truncated protein and loss of function.”

In 2.1.1 Case presentation, the gene accession number was erroneously written as “NM_4560.4”.

A correction has been made to section 2 Case Presentation, 2.1 Proband 1, 2.1.1 Case presentation, Paragraph 1]:

“The proband was a 32-year-old man referred to our clinic due to a two-year history of primary infertility with his wife who was diagnosed with dwarfism at 12 years old and later was found to harbor a novel variant (c.1133T>C (p.F378S) (NM_004560.4)) in ROR2 gene presenting at homozygous state with her distinctive features (hypertelorism, prominent eyes, wide palpebral fissures, broad and depressed nasal bridge, short upturned nose, anteverted nares, tented upper lip, broad and triangular mouth, brachydactyly, hypoplastic nails, and fifth finger clinodactyly) consistent with Robinow syndrome. Family-based segregation analysis showed that both his wife’s siblings and her parents were ROR2 c.1133T>C heterozygous carriers. Since his wife desired pregnancy and came to our hospital, we recommended that her husband (the proband) undergo a comprehensive examination in our hospital.”

The original version has been updated.

